# Nitrogen Dioxide Sterilization in Low-Resource Environments: A Feasibility Study

**DOI:** 10.1371/journal.pone.0130043

**Published:** 2015-06-22

**Authors:** Majdi Shomali, David Opie, Trisha Avasthi, Ariel Trilling

**Affiliations:** 1 Eniware LLC, Washington D.C., United States of America; 2 Noxilizer Inc., Baltimore, Maryland, United States of America; Chang Gung University, TAIWAN

## Abstract

Access to sterilization is a critical need for global healthcare, as it is one of the prerequisites for safe surgical care. Lack of sterilization capability has driven up healthcare infection rates as well as limited access to healthcare, especially in low-resource environments. Sterilization technology has for the most part been static and none of the established sterilization methods has been so far successfully adapted for use in low-resource environments on a large scale. It is evident that healthcare facilities in low-resource settings require reliable, deployable, durable, affordable, easily operable sterilization equipment that can operate independently of scarce resources. Recently commercialized nitrogen dioxide (NO_2_) sterilization technology was analyzed and adapted into a form factor suitable for use in low-resource environments. Lab testing was conducted in microbiological testing facilities simulating low-resource environments and in accordance with the requirements of the international sterilization standard ANSI/AAMI/ISO 14937 to assess effectiveness of the device and process. The feasibility of a portable sterilizer based on nitrogen dioxide has been demonstrated, showing that sterilization of medical instruments can occur in a form factor suitable for use in low-resource environments. If developed and deployed, NO_2_ sterilization technology will have the twin benefits of reducing healthcare acquired infections and limiting a major constraint for access to surgical care on a global scale. Additional benefits are achieved in reducing costs and biohazard waste generated by current health care initiatives that rely primarily on disposable kits, increasing the effectiveness and outreach of these initiatives.

## Introduction

Access to sterilization equipment is necessary to reduce disease transmission from instruments during surgical procedures. Lack of access to sterilization limits access to surgery, contributing to the fact that less than four percent of the world’s surgical procedures are performed in countries with limited resources.[1] As a result, there are an estimated five billion people worldwide who do not receive essential surgical care. A recent Lancet commission concluded that in 2010, 33 percent of all deaths worldwide were caused by conditions that could have been treated with surgery.[2] In 2008 alone, an estimated 3.1 million women in 49 developing countries suffered from obstructed labor, a condition that can be averted by Cesarean section.[3] The WHO recommends the autoclave as the primary method of sterilization for surgical care at the district hospital level, but lack of electricity, maintenance requirements, and high prices for sterilization equipment have made implementing these recommendations extremely difficult.[4] For example, 66 percent of healthcare facilities in sub-Saharan Africa do not have reliable access to electricity.[5] Surgical wards in low- and middle-income countries report infection rates as high as 46 percent, due in part to lack of access to surgical instrument sterilization.[6] Inadequate sterilization may also contribute to the spread of HIV and other diseases that are transmitted through contact with bodily fluid. Therefore, quality of care in health facilities and population health can be greatly improved by introducing the capability to sterilize instruments in a form factor suited for these environments.

In the absence of viable sterilization equipment and processes, global healthcare initiatives have resorted to the use of disposable kits. These kits do not take into account the added overhead expense that significantly increases the true cost of this approach.[7] These additional costs limit the reach of these initiatives. Furthermore, this approach creates a new challenge by generating large quantities of biomedical waste in areas that are not equipped to handle their proper disposal and does not strengthen healthcare systems.[7]

It is evident that healthcare facilities in low-resource settings require reliable, deployable, durable, affordable, easily operable sterilization equipment that can operate independently of scarce resources. The use of nitrogen dioxide (NO_2_) as a sterilant offers to address this need by enabling a portable gas sterilization system that does not rely on electrical power.

### Introduction to NO_2_ Sterilization

Nitrogen dioxide is a sterilant gas that is used in the terminal sterilization of medical instruments. The NO_2_ sterilization process has been validated for the terminal sterilization of medical devices using typical sterilization chamber-based systems. There are unique advantages with the NO_2_ process that are not available with other sterilant gases. These advantages include operation at room temperature, a relatively low sterilant concentration, rapid microbicidal activity, and minimal sterilant residuals on processed articles. These advantages permit the use of the NO_2_ process in low-resource environments.

Nitrogen dioxide is a gas at room temperature, not a vapor, permitting rapid penetration into packages and challenging locations in the medical device loads. The boiling point of NO_2_ is 21^°^C at sea level, which results in a relatively high (101,325 Pa) saturated vapor pressure at ambient temperature. Additionally, the NO_2_ process requires a concentration of about one percent of ambient pressure gas, which is relatively low compared to other gas sterilants. Because of this, the concentration of NO_2_ used during a sterilization cycle is far below the NO_2_ dew point, and condensation of the sterilant will not occur. Also, this gaseous nature of NO_2_ at ambient conditions allows for efficient aeration of the load, making the processed articles safe to handle.[8]

A sterilant is a substance that inactivates all forms of microorganisms, including bacteria, bacterial spores, fungi, fungal spores, and viruses. The microorganisms tested to demonstrate microbicidal efficacy are listed in Table 1. The listed microorganisms are those recommended for testing in the applicable regulatory documents.[9,10] All of these microorganisms exhibited rapid lethality upon exposure to NO_2_. However, *Geobacillus stearothermophilus* exhibits higher resistance to the NO_2_ process.

**Table 1:** List of Microorganism Type and Reference Organism Tested.

Organism Type	Reference Organism Tested
Bacterial Spores	*Geobacillus stearothermophilus* (ATCC 7953) *Bacillus atropheus* (ATCC 9372) *Bacillus pumilus* (ATCC 27142) *Clostridium sporogenes* (ATCC 3584) *Bacillus subtilis* var. *niger* (ATCC 49278)
Vegetative Spores	*Psuedomonas aeruginosa* (ATCC 27559) *Salmonella enterica* ssp. *Typhimurin* (ATCC 14028) *Staphylococcus aureus* (ATCC 6538)
Mycobacteria	*Mycobaterium terrae* (ATCC 15755)
Fungi	*Tricophyton mentagrophytes* (ATCC 18748) *Candida Albicans* (ATCC 10231)
Non-lipid Viruses	*Porcine parvovirus*
Lipid Viruses	*Herpes simplex virus* Type 1

A biological indicator (BI) is used to monitor the effectiveness of the sterilization process. A BI consists of a known population of the highly resistant microorganism (usually spores), inoculated onto a suitable carrier and packaged. This indicator organism should be more resistant to the NO_2_ process than the bioburden found on medical devices. For the NO_2_ process, *G*. *stearothermophilus* was identified as the most resistant organism and used as the indicator organism in all trials.

The microbicidal mechanism of action for NO_2_ has been identified as single-strand breaks in DNA. Studies of DNA degradation have included exposing and evaluating purified DNA (removed from the microorganism prior to exposure), as well as evaluating DNA that was extracted from exposed, intact organisms.[11–13] Furthermore, it has been observed that the occurrence of DNA single-strand breaks increase with the increasing NO_2_ exposure duration.

The NO_2_ process is compatible with most medical device materials. Materials that are compatible with NO_2_ are shown in Table 2. Materials that are not compatible, as well as compatible alternative materials, are shown in Table 3. Sterile barrier packaging that may be used with the NO_2_ process include non-woven, polypropylene surgical wraps and Tyvek pouches. Paper (cellulosic) packaging is not compatible.

**Table 2:** Partial List of Materials That Are Compatible With the NO_2_ Process.

Stainless Steel	Polyethylene	Polyetherimide
Anodized Aluminum	Polypropylene	Polycarbonate
Gold (Plating)	PET / PETG	Cyclic Olefins
Glass / Ceramic	Polystyrene	PVC^a^
Fluoropolymers	Polysulfones	Silicone^a^
Viton (Gaskets)	PEEK / PAEK	Hypalon

^a^ Depends on grade of material

**Table 3:** List of Materials That Are Not Compatible With the NO_2_ Process and Alternative Materials.

Incompatible Material	Design Alternatives
Polyurethane	Thermoplastic Elastomers (TPE)
Nylon	Polyester, Polyolefin
Delrin (polyacetal)	PEEK, PSU, PEI
Cellulose-based (some paper)	Polyester or styrene label stock

For the materials that are compatible with the NO_2_ process, biocompatibility of the exposed medical devices has been demonstrated. For the majority of materials, cytotoxicity was used as a screening tool for biocompatibility of the materials listed in Table 2. In these tests, none of the materials exposed exhibited an increase in cytotoxic response after exposure to the NO_2_ process. For devices validated with the NO_2_ process, the materials of construction were tested for biocompatibility according to ISO 10993 methodology.[14] This testing has shown that the NO_2_ does not leave harmful (bio-incompatible) residuals.

A successful sterilization technology for use in low-resource environments must meet four general requirements: electrical power-free operation, sterilization efficacy, operator safety, and the preservation of medical instrument functionality. The first requirement was implemented by design, with the development of a portable sterilizer that does not require electricity. The testing of the other three requirements is reported herein.

Sterilization of medical devices in a low-resource environment requires a sterilizer specifically designed for this application. Where a low-resource environment has non-existent or unreliable electrical service, room temperature and ambient pressure operation of the sterilization process is essential. Heating the load in the sterilizer and using vacuum pumps, commonly required with most sterilization processes, are not possible in low-resource environments. Therefore, the room-temperature and ambient pressure operation of the NO_2_ process is the property that enables the portable sterilizer.

**Fig 1:**
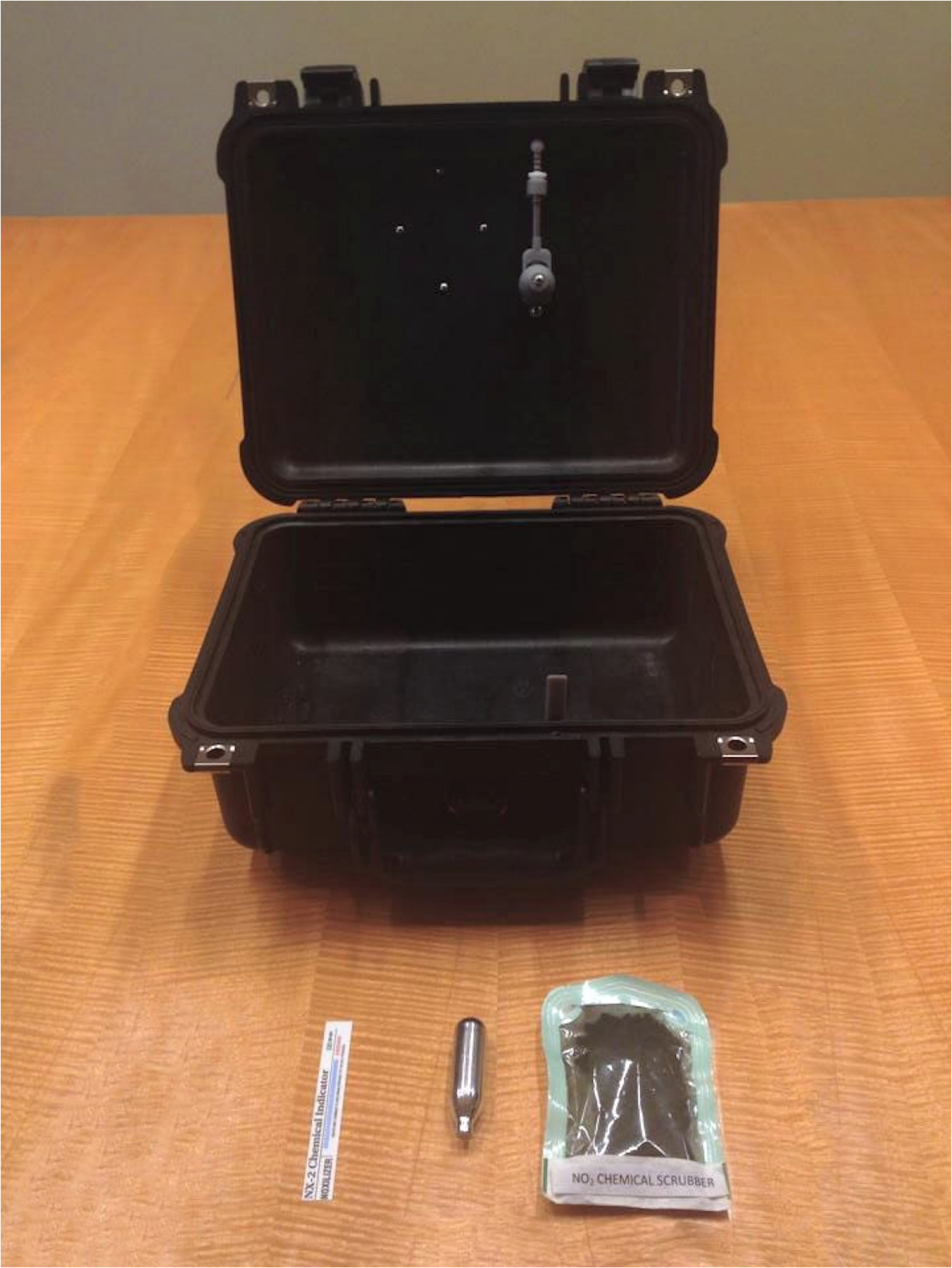
The Portable Sterilizer with Consumables. The portable sterilizer consists of a lockable case, a cartridge of sterilant gas, and an absorbent scrubber. A chemical indicator confirms successful sterilization.

This portable sterilizer (Fig 1) consists of a lockable case made from polypropylene polymer, supplied with sterilizing gas in a miniature cartridge and a gas scrubber. In this form, the portable sterilizer is an easy to use, comprehensive system in which medical devices can be sterilized. The design of the case and the gas delivery module allow for operation without a power source. Instruments are exposed to the sterilant gas through passive diffusion once the gas is mechanically released from the cartridge into the case. Absorption of the gas to a level that is safe for human exposure also occurs passively through the scrubber medium. A battery-operated timer tracks the sterilization and absorption process, alerting the operator when the cycle is complete and the case can be opened. Electricity is not used for any step of the process. All of the testing reported here used this version of sterilizer.

To use the portable sterilizer, medical devices are washed and dried. Optionally, the medical devices can be packaged in preparation for the sterilization process. While loading the portable sterilizer, the operator places the medical devices in the portable sterilizer along with a package of sterilant-absorbing media (the scrubber). The sterilant cartridge is placed in the sterilant compartment. The act of closing and locking the enclosure releases the sterilant into the case and starts the sterilization process. The sterilant-absorbing media will immediately begin to absorb the sterilant. But, because the rate of sterilant absorption is much slower than the rate of sterilant diffusion within the case, the entire contents of the case are exposed to the sterilizing gas for a duration that is sufficient to sterilize the medical devices.

The medical device articles selected for testing provide the greatest challenge to penetration of the sterilant. The device configurations that pose the greatest challenge to the penetration of sterilant are lumens, mated surfaces and hinges. The inocula must be placed in various locations on the test articles including those least favorable to penetration and contact with the sterilant, e.g., lumens, mated surfaces, and hinges.[9]

## Methods

The sterilizing enclosure used for the tests reported here had an internal volume of nine liters and weighs less than one kg. A rack was included for holding the articles to be processed. Gas connections enabled measuring the environment inside the portable sterilizer enclosure (hereafter, ‘enclosure’) during the sterilization cycle. The concentration of NO_2_ sterilant and humidity in the enclosure was measured with a Fourier transform infrared spectroscopy (FTIR) system (California Analytical Instruments model 600 SC FTIR with 50°C gas cell temperature). This system used a sampling pump to sample air within the enclosure, circulate the gas through the optical gas cell of the FTIR, and then return the gas back to the enclosure. The rate of gas sampling was sufficiently low in order to not perturb the operating conditions. The FTIR system recorded the NO_2_ concentration and humidity once every 20 seconds.

Sterilization efficacy was tested by inoculating surgical instruments in a most-challenging location, packaging the instruments, and then exposing the instruments to the NO_2_ process. The most-challenging location is determined to be the gap between segments of hinged medical devices. For testing here, the hinged portion of standard stainless steel Mayo scissors and hemostatic forceps were used.

A spore suspension of *G*. *stearothermophilus* (ATCC 9573 strain) was used to inoculate the hinged devices. Each of these hinged devices was opened to expose the hinge portion of the device and the spore inoculum was placed in the hinged portion of the scissors and forceps. The hinge mechanism was actuated (opened and closed) several times to ensure that the spores were between the mated surfaces of the hinged device. Inoculum was also applied to other surfaces of the stainless steel devices so that the total inoculum on each device was more than 10^6^ spores of *G*. *stearothermophilis*. Control samples were used to verify the inoculated population of spores. The inoculum on each device was allowed to air dry for no less than 30 minutes prior to further processing. BIs were included in the cycles and each BI had more than 10^6^ spores of *G*. *stearothermophilus* on a stainless steel carrier. Each BI was packaged in a Tyvek and Mylar pouch.

The exposure cycle parameters are summarized in Table 4. Three inoculated and packaged hinge samples and three packaged BIs were included in the enclosure for each cycle. The sterilant dose was applied to the enclosure using either one of two methods. The first method adds a small amount of liquid NO_2_ (in the liquid state, NO_2_ forms the dimer N_2_O_4_). This liquid boils quickly at room temperature, with the amount of liquid added being sufficient to yield 10,000 ppm to 14,000 ppm of NO_2_ gas in the enclosure. Alternatively, the NO_2_ dose was added to the EPS using a chemical reaction between two compounds which resulted in the evolution of NO_2_ as the chemical reaction by-product. Both methods resulted in similar NO_2_ doses.

**Table 4:** Table Summarizing Cycles Completed in the Portable Sterilizer.

Cycle Data Code	Peak NO_2_ Conc. (ppm)	Relative Humidity (%)	Temp. (°C)	Dwell Time (Minutes)	Hinge Devices	BIs
420140926B	13350	22	21.5	32	3	3
420140929B	11540	45	22.0	32	3	3
420140923A	17870	43	23.0	15	3	3
420140923B	18510	24	24.3	15	3	3
420140930A	14610	29	21.0	(Passive scrubbing)	3	3
420141001A	13570	43	21.2	(Passive scrubbing)	3	3
420141002A	705	57	21.8	(Passive scrubbing)	3	3

The humidity was varied for the cycles completed in order to represent the range of relative humidity in which the portable sterilizer will be used. The range of initial relative humidity, at the start of the cycle, was between 22 percent and 57 percent relative humidity. The control of the humidity was achieved by placing the sterilizing enclosure in an enclosed environment and using dry or humidified air to achieve the target value. Higher humidity facilitates faster lethality with the NO_2_ process. Relative humidity values used in these tests are relatively low, increasing the challenge for the NO_2_ process.

Aeration was completed using either of two methods: passive or active scrubbing. Passive scrubbing was completed by placing the two Tyvek pouches in the enclosure, with each pouch containing 18.7 grams of sodium permanganate scrubber media (Purafil SP Media). Active scrubbing used a manual pump to draw fresh air into the EPS. The exhausted air passed through the Purafil scrubber media to remove the NO_2_. The purpose of the active scrubbing was to halt the sterilizing action of the NO_2_ for the purpose of evaluating sub-lethal microbicidal results of shortened cycles on the BIs and hinge devices.

After exposure, the hinges and Tyvek/Mylar pouches were inspected for evidence of incompatibility with the NO_2_ process. Upon removal from the Tyvek/Mylar pouch, each hinged sample and BI was aseptically transferred to a tube containing 10 ml tryptic soy broth (TSB) and then incubated at 55–60°C. After seven days of incubation, the tubes were evaluated for evidence of growth. The FTIR data was analyzed to ensure the target sterilant concentration and humidity was achieved in each cycle.

## Results and Discussion

The results of cycle efficacy testing are shown in Table 5. For each hinge device and BI, the test tubes containing TSB and hinges or BIs were evaluated for growth after seven days. TSB media that becomes turbid is considered ‘positive’, indicating that the hinged device or BI was not sterile after the process. All of the inoculated hinge devices were sterile after each cycle tested. However, Table 5 shows that some of the cycles terminated with active scrubbing had surviving BIs.

**Table 5:** Table of Biological Indicator Results.

Cycle	BI (# positive/Number tested)	Hinges (# positive/Number tested)
420140926B	1/3	0/3
420140929B	1/3	0/3
420140923A	0/3	0/3
420140923B	0/3	0/3
420140930A	0/3	0/3
420141001A	0/3	0/3
420141002A	3/3	0/3

The surviving BIs result from two factors. The first factor is that the BI has 10^6^ spores on a 7.0 mm diameter stainless steel disc. This high density of spores on the BI results in some clumping of the spores as the BI dries. The clumping increases the resistance to the sterilization process. The second factor to consider is that the positive BIs were exposed in truncated cycles with the lowest NO_2_ concentration levels. Cycle development and validation will leverage these results to reach a validated cycle for stainless steel surgical instruments.

**Fig 2:**
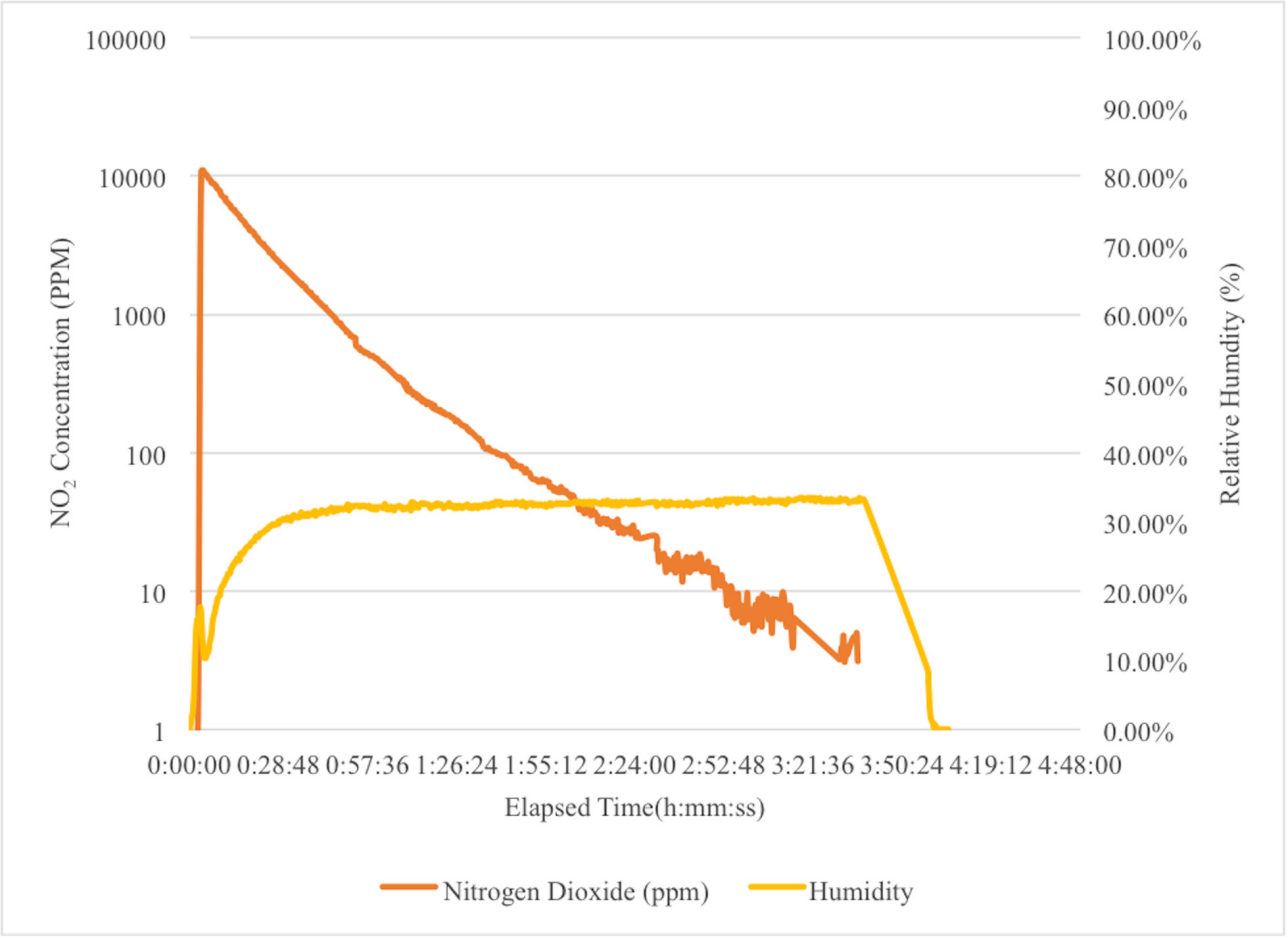
The Sterilization Cycle With Passive Aeration. After dosing with NO_2_, the sterilant is absorbed by the scrubber media in the case. The cycle ends when the sterilant concentration has returned to a safe level.

The NO_2_ dose with passive aeration is shown in Fig 2. The graph shows the initial relative humidity, followed by the dosing with NO_2_, and then absorption of the sterilant by the scrubber media. The cycle ends when the sterilant level returns to a safe level, which requires between three to eight hours to complete, depending on the exposure requirements of the cycle. Sterility of the instruments is maintained as long as the case remains closed, so the sterilization cycle can be left to run overnight.

**Fig 3:**
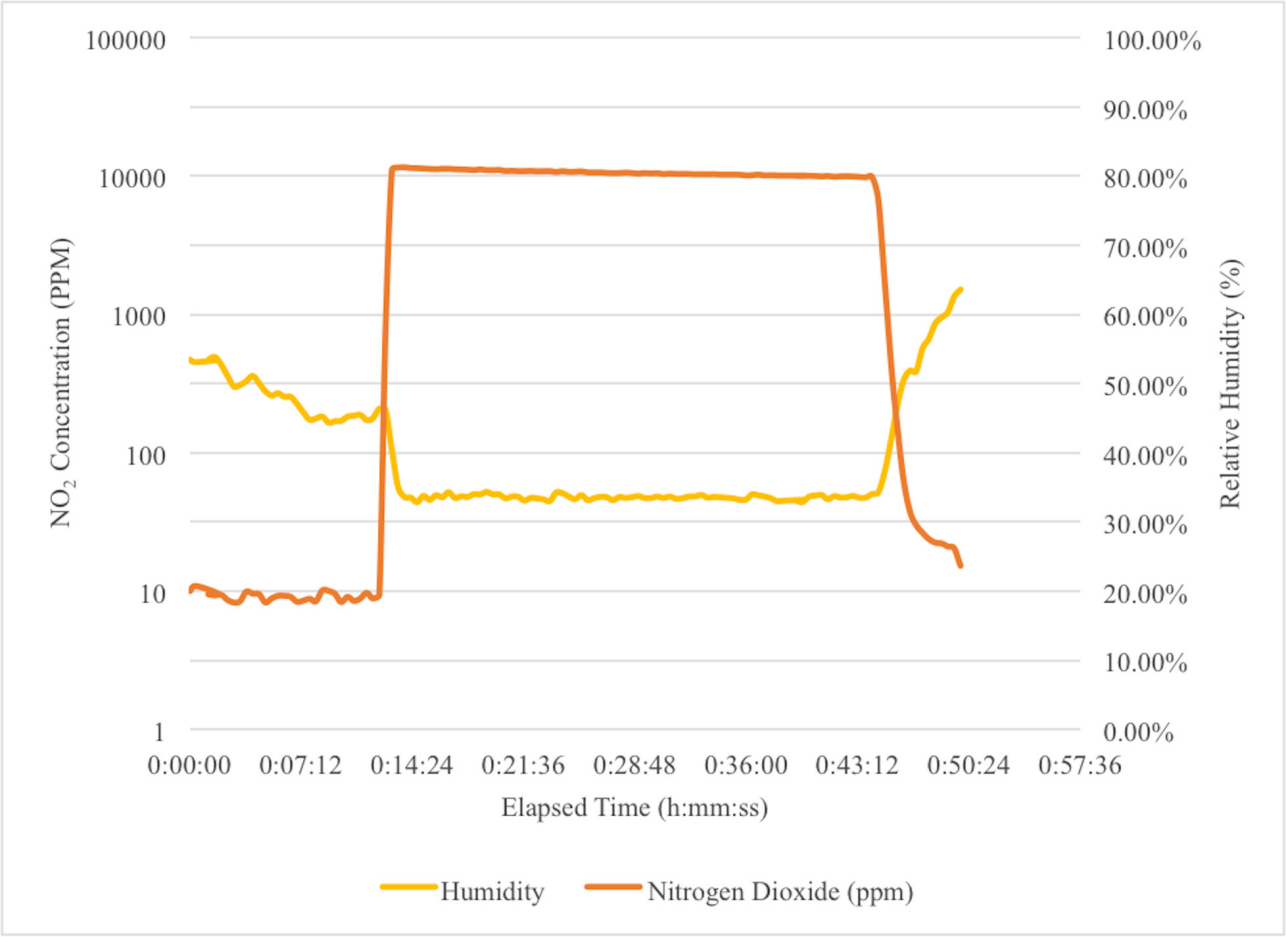
The Sterilization Cycle With Active Aeration. After dosing with NO_2_ and a dwell period, aeration is initiated and the NO_2_ is removed.

The NO_2_ dose and humidity in the enclosure during the sterilization cycle for a typical cycle with active scrubbing was measured with the FTIR and is shown in Fig 3. This graph shows the humidity and NO_2_ concentration. The humidity decreases with the addition of dry air until the target humidity is reached. Then, the NO_2_ is added to the enclosure. After a dwell period, aeration begins and the NO_2_ is removed.

All of the packages and hinged devices were inspected after exposure. None of the packages or stainless steel hinged devices exhibited any material change due to the exposure to the NO_2_ process. These observations agree with material testing completed on medical device materials.[15]

## Financial Considerations

Affordability is a key requirement for widespread adoption of this technology. An analysis of the costs associated with this form factor established the price of the sterilizing enclosure to be approximately US$500. In addition to the sterilizing enclosure, a set of consumables estimated to cost US$5 for each sterilization cycle. With these estimates, a financial analysis was conducted to analyze a cost comparison of a Voluntary Medical Male Circumcision (VMMC) healthcare initiative using prepackaged kits with disposable surgical instruments versus reusable instruments with sterilization capability using the portable sterilizer form factor. The focus was placed on costs that are dependent on the approach used for the initiative, and not costs that were independent of the approach such as infrastructure and counseling. Use of reusable instruments along with sterilization capability would reduce the estimated process dependent costs of US$85.17 per procedure to an estimated cost of US$5.73–7.30 per procedure.[7] The details of this analysis are shown in Table 6.

**Table 6:** Cost Analysis of VMMC Program: Using Sterilization vs Disposable Kits^*^.

	VMMC Disposable Kit with Disposable Surgical Instruments (US$)	Equivalent VMMC Kit with Reusable Instruments and Sterilization Using Portable Sterilizer – est. 100 VMMC/kit (US$)
VMMC Kit	$25.17 (pre-sterilized kit using ethylene oxide, contains disposable instruments and consumables)	$0.20 - $0.23 (Instruments only, higher quality suitable for re-use and re-sterilization, cost $20–23, estimated use 100 cycles, cost per cycle is 1% of procurement cost)
Supplementary consumable Supplies per VMMC (gauze, scalpel, syringe with needle, gloves, apron, sutures, surgical tape, alcohol swabs, and an O drape)	Included in above kit	$4.13 - $5.07
Supply chain and waste management overhead cost	$60.00	$0.60-$1.20 (estimated 1–2%)
Biologically contaminated waste per VMMC	0.5 kg	0.05 kg
Sterilization Cost	Included in above kit	$0.80 (estimated 20 kits can be sterilized per cycle, includes labor, consumables for sterilization cycle, amortization of cost of sterilizer over usable lifetime and the cost of a Tyvek pouch)
Additional Overhead Costs	Not quantified by study	Expected to be 1–2% of overhead that is incurred with disposable kits
Cost per VMMC	$85.17, plus overhead and 0.5 kg biologically contaminated waste	$5.73-$7.30, plus reduced overhead and reduced biologically contaminated waste per cycle

* Not a comprehensive cost analysis, does not include process independent healthcare infrastructure and counseling costs, intended to show where approaches differ

## Conclusions

The technical feasibility of the portable sterilizer has been demonstrated, showing that sterilization of medical instruments can occur without using electrical power. This small, portable sterilizer is well suited for low-resource environments and can sterilize challenging medical devices. Additionally, the sterilant can be passively absorbed, permitting safe retrieval of the sterile products at the end of the sterilization cycle.

Future studies will demonstrate the practical feasibility of the portable sterilizer in low-resource settings through testing in the field. Several safety mechanisms are included in the sterilizer design to eliminate the possibility of operator exposure to harmful levels of sterilant gas. The operator is unable to initiate a sterilization cycle prior to closing the enclosure, and is prevented from opening the enclosure until the timer indicates the conclusion of the cycle. To ensure proper use of the sterilizer, an operator training program will be designed and is scheduled to be validated by an independent partner with extensive experience in health program implementation (Jhpiego). Operator training will consist of a direct hands-on training session. Additionally, safe operation and trouble-shooting of the sterilizer will be described in a user manual using both text and images.

The technology is affordable, scalable, and compatible with a wide range of materials. It also represents a major cost savings compared to the fully disposable kit model currently used by some healthcare initiatives enabling them to be more effective and extend their reach. Additional benefits can also be realized by establishing sterilization capability in the target low-resource environments and thereby improving their healthcare systems.
